# Cerebellar Long Noncoding RNA Expression Profile in a Niemann-Pick C Disease Mouse Model

**DOI:** 10.1007/s12035-021-02526-3

**Published:** 2021-08-19

**Authors:** Shiqian Han, Meng Ren, Tianyin Kuang, Mao Pang, Dongwei Guan, Yesong Liu, Yong Wang, Wengeng Zhang, Zhijia Ye

**Affiliations:** 1 Department of Tropical Medicine, College of Preventive Medicine, Army Medical University (Third Military Medical University), Chongqing, 400038 China; 2 Laboratory Animal Research Center, Chongqing University School of Medicine, Chongqing, 400044 China; 3 Cornell University, Ithaca, NY 14853 USA; 4 Department of Laboratory Animal Science, College of Basic Medical Sciences, Army Medical University (Third Military Medical University), Chongqing, 400038 China; 5 Precision Medicine Key Laboratory of Sichuan Province and Precision Medicine Center, West China Hospital, Sichuan University, Chengdu, 610041 China

**Keywords:** Niemann-Pick type C disease, Cerebellum, Long noncoding RNAs, Coexpression network, LncRNA H19

## Abstract

Niemann-Pick type C (NP-C) disease is a neurodegenerative lysosomal storage disorder primarily caused by mutations in *NPC1*. However, its pathogenesis remains poorly understood. While mounting evidence has demonstrated the involvement of long noncoding RNAs (lncRNAs) in the pathogenesis of neurodegenerative disorders, the lncRNA expression profile in NP-C has not been determined. Here, we used RNA-seq analysis to determine lncRNA and mRNA expression profiles of the cerebella of NPC1^−/−^ mice. We found that 272 lncRNAs and 856 mRNAs were significantly dysregulated in NPC1^−/−^ mice relative to controls (≥ 2.0-fold, *p* < 0.05). Quantitative real-time PCR (qRT‐PCR) was utilized to validate the expression of selected lncRNAs and mRNAs. Next, a lncRNA-mRNA coexpression network was employed to examine the potential roles of the differentially expressed (DE) lncRNAs. Functional analysis revealed that mRNAs coexpressed with lncRNAs are mainly linked to immune system–related processes and neuroinflammation. Moreover, knockdown of the lncRNA H19 ameliorated changes in ROS levels and cell viability and suppressed the lipopolysaccharide (LPS)–induced inflammatory response in vitro. Our findings indicate that dysregulated lncRNA expression patterns are associated with NP-C pathogenesis and offer insight into the development of novel therapeutics based on lncRNAs.

**Supplementary Information:**

The online version contains supplementary material available at 10.1007/s12035-021-02526-3.

## Introduction

Niemann-Pick type C (NP-C) disease is a rare, autosomal recessive, neurodegenerative lysosomal disorder. Approximately 95% and 5% of NP-C cases are caused by mutations in *NPC1* and *NPC2*, respectively [1]. The NPC2 protein binds cholesterol released from low-density lipoprotein (LDL) in the lysosome (Lys) lumen and delivers it to the Lys membrane-spanning protein NPC1, which facilitates cholesterol transport to the endoplasmic reticulum and plasma membrane [2–4]. Consequently, *NPC1* or *NPC2* deficiency causes the accumulation of massive amounts of unesterified cholesterol and other lipids (especially glycosphingolipids) in late endosomes (LEs)/Lys [5]. NP-C is characterized by various pathological features in the cerebellum, including the loss of Purkinje cells [6–8]. Its clinical manifestations include progressive neurodegeneration characterized by cerebellar ataxia, dementia, dysphagia, vertical gaze palsy, and gelastic cataplexy [8]. The clinical manifestations and progression of NP-C can be heterogeneous; once neurodegenerative decline has begun, the disease is fatal [1]. To date, how NPC1 protein deficiency impairs brain function, leading to dementia and neurodegeneration, remains unclear. Moreover, clinical treatment agents for NP-C are limited [9]. Thus, a comprehensive understanding of the mechanisms underlying NP-C pathogenesis is urgently required for the development of novel effective therapies.

Long noncoding RNAs (lncRNAs) are comprised of RNA molecules greater than 200 bases and generally lack protein-coding function [10]. LncRNAs play critical regulatory roles in various biological processes, including genomic imprinting, transcriptional and posttranslational regulation, the maintenance of stem cell pluripotency and the immune response [11]. Numerous studies have implicated dysregulated lncRNAs in neurodegenerative disorders such as Alzheimer’s disease [12, 13], autism spectrum disorder [14], Parkinson’s disease [15], and Huntington’s disease [16], and because of their emerging roles as key modulators of neurodevelopmental pathogenesis, lncRNAs have potential applications as biomarkers or therapeutic targets against neurological disorders [17, 18]. However, little about lncRNAs and their functional implications in NP-C has been determined.

Here, we used RNA-seq to determine differentially expressed lncRNAs and mRNAs in the cerebella of NPC1^nih^ mice, a well-characterized animal model of NP-C. A lncRNA-mRNA coexpression network was created, and Gene Ontology (GO) and Kyoto Encyclopedia of Genes and Genomes (KEGG) analyses were carried out to predict lncRNA function. Additionally, a NPC1-related coexpression network was constructed to elucidate functional interactions between lncRNAs and NPC1, and the possible mechanisms underlying the development of NP-C were examined.

## Materials and Methods

### Animal Models

NPC1^nih^ (NPC1^−/−^, KO) mice and wild-type (WT) mice were bred from heterozygous pairs of BALB/cNctr-Npc1m1N/J mice (The Jackson Laboratory). The mice were maintained in rooms at a controlled temperature (22–24°C) and humidity (40–60%) under a 12-h light/dark cycle. Mouse weight was monitored weekly, and survival time was recorded. Rotarod and coat hanger tests were performed as previously described [19, 20], with a minor alteration to the rotarod test; the mice were allowed to remain on the platform for a maximum of 100 s. The cerebella of male NPC1^−/−^ mice and WT controls at 7 weeks of age were used for RNA-seq analysis.

### Mouse Neurobehavioral Assessment

Neurobehavioral assessment was performed as described previously [21]. The assessment involved ledge, hind limb-clasping, gait, and kyphosis tests. Each measurement was assessed by scoring on a scale of 0–3 (0 indicating no phenotype, 1 indicating a weak phenotype, 2 indicating a strong phenotype, and 3 indicating the most severe phenotype), with a total score ranging from 0 to 12.

### RNA Extraction and RNA-seq Analysis

Total RNA was isolated from mouse cerebellar tissue using TRIzol reagent (Invitrogen). RNA integrity was determined on an Agilent 2100 Bioanalyzer (Agilent Technologies). RNA purity was determined using a NanoPhotometer spectrophotometer (Implen), and RNA concentration was assessed using a Qubit RNA Assay Kit on a Qubit 2.0 fluorometer (Life Technologies). The RNA samples were then used for RNA-seq and qRT-PCR analyses.

RNA-seq analysis was performed as described previously [22]. Library construction and RNA-seq were performed by Genedenovo Biotechnology Co., Ltd. (Guangzhou, China). The RNA-seq data were normalized based on fragment per kilobase of transcript per million mapped reads (FPKM) by StringTie [23]. DESeq2 program was applied to analyze the differential expression genes between two different groups [24]. Differently expressed (DE) transcripts with a fold change ≥ 2 and *p* < 0.05 in expression were identified by comparison.

### qRT-PCR Validation

qRT-PCR analysis was performed on a CFX96 Real-Time PCR system with the following conditions: 95 °C for 3 min, followed by 40 cycles of 95 °C for 5 s and 60 °C for 30 s. Relative lncRNA and mRNA expression were determined using the 2^−△△Ct^ approach. *GAPDH* with no differential expression between two groups in the RNA-Seq profile (Table S1) was used as the reference gene. The primer sequences are provided in Table S2.

### GO and KEGG Pathway Analyses

GO annotation and KEGG pathway analyses were performed to determine the roles of all identified DE mRNAs. GO analysis, which was conducted to annotate the attributes of the genes and gene products, was used to examine enrichment of 3 types of terms in the DE mRNAs: biological process, molecular function, and cellular component terms (http://www.geneontology.org). Pathway analysis was used to determine the biological pathways enriched in the DE genes (http://www.genome.jp/kegg/).

### Construction of a Coexpression Network

To determine the relationships between DE lncRNAs and mRNAs, a coexpression network was built based on the results of correlation assessment of DE lncRNAs and mRNAs [25]. A Pearson’s correlation coefficient ≥ 0.980 and *p* < 0.05 were used as cutoffs to determine the DE lncRNAs and mRNAs. To examine interactions between NPC1 and the lncRNAs and to predict the functions of related lncRNAs, we identified NPC1-related coexpression networks and used Cytoscape (The Cytoscape Consortium) for visualization.

### Small Interfering RNA Transfection

Skin fibroblasts were isolated from 7-week-old WT and NPC1^−/−^ mice as previously described [26]. Small interfering RNA (siRNA) against mouse H19 (5′-GCAGAATGGCACATAGAAA-3’) and control siRNA were synthesized by RiboBio (Guangzhou, China). The skin fibroblasts were transfected with 50 nM si-H19 or control siRNA by electroporation using a NEPA21 electroporator (Nepa Gene) according to the manufacturer’s recommendations. Forty-eight hours after electroporation, the cells were harvested and used in the following assays.

### Cell Viability Assay

Cell viability was assessed using the Cell Counting Kit-8 (CCK-8, Biyuntian Biotechnology, Jiangsu, China) assay. Mouse skin fibroblasts were seeded into 96-well microplates at a density of 5 × 10^3^ cells/well after electroporation. Cell viability was assessed by incubating each well with 100 µL of CCK-8 solution for 4 h after 48 h of culture under the designated conditions (37 °C and 5% CO_2_), and the absorbance at 450 nm was measured.

### Measurement of Intracellular ROS

Intracellular ROS were detected using the cell-permeant dye CM-H_2_DCFDA (Invitrogen). At 48 h after electroporation, fibroblasts were trypsinized, incubated with 5 μmol/L CM-H_2_DCFDA for 30 min at 37 °C, and washed twice with PBS. Subsequently, the intracellular formation of ROS was determined by measuring fluorescence with 488-nm excitation and 538-nm emission wavelengths using a BD Accuri C6 flow cytometer. Relative ROS levels are expressed as the mean fluorescence intensity.

### Lipopolysaccharide-Induced Inflammation Assay

Mouse skin fibroblasts after electroporation were seeded at 5 × 10^4^ cells/well in 12-well plates to incubate for 48 h, and then the cells were stimulated with 100 ng/mL lipopolysaccharide (LPS) for 24 h to trigger an inflammatory reaction and collected for RT-PCR.

### Statistical Analyses

All data are indicated as the mean ± standard error of the mean (SEM). Student’s *t*-test was employed to compare the control and experimental groups. One-way analysis of variance (ANOVA) was used to analyze three or more groups. Statistical analyses were done using SPSS 20.0. *p* < 0.05 indicated statistical significance.

## Results

### NPC1^−/−^ Mice Showed Cerebellar Phenotypes

**Fig. 1:**
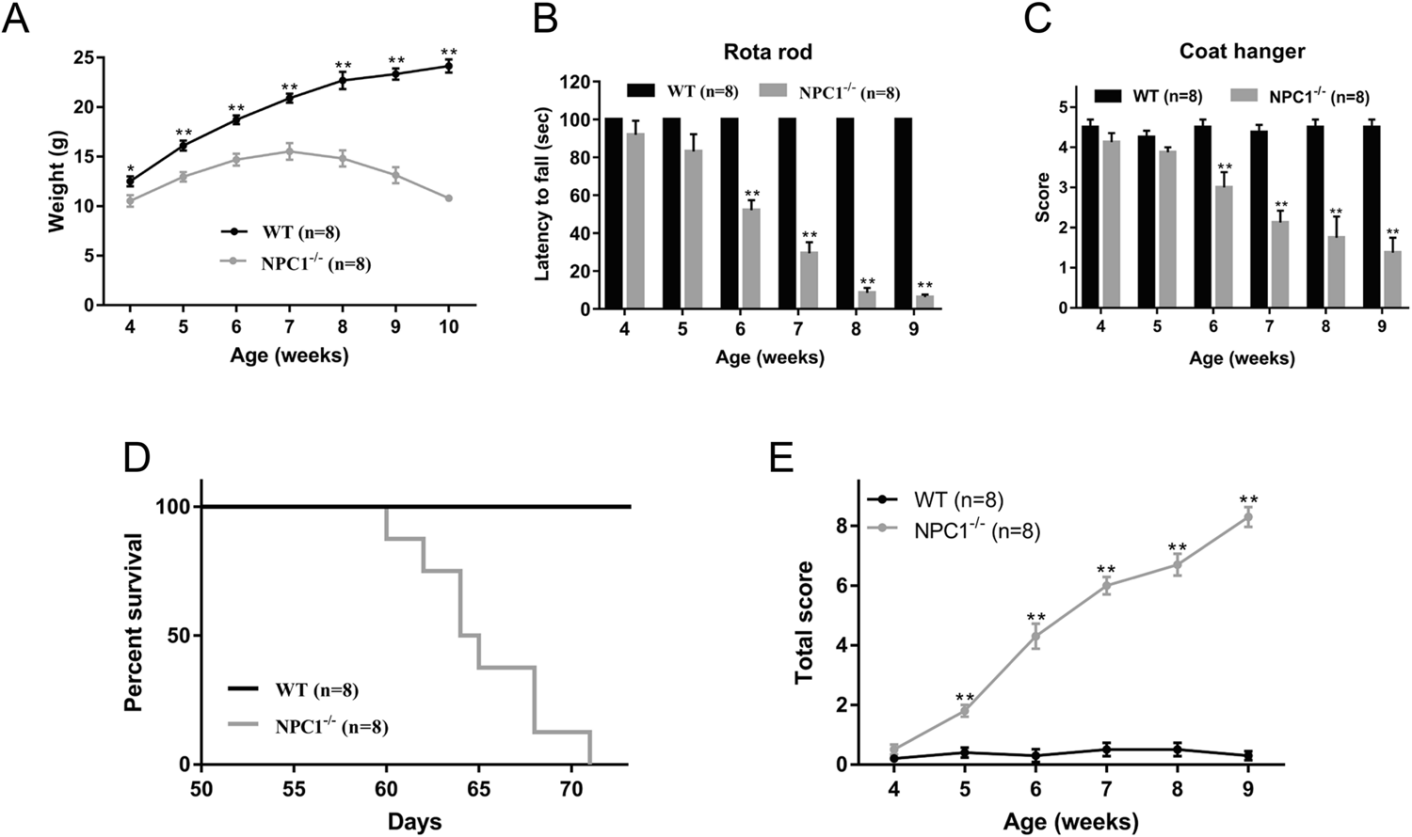
Cerebellar phenotypes following *NPC1* knockout. **A** Comparison of the body weights of WT and NPC1^−/−^ mice. **B** Rotarod performance of WT and NPC1^−/−^ mice. **C** Coat hanger assessment of WT and NPC1^−/−^ mice. **D** Survival analysis of WT and NPC1^−/−^ mice. **E** Composite phenotype assessment of WT and NPC1^−/−^ mice. The NPC1^−/−^ mice exhibited a progressive phenotype that was markedly different from the corresponding phenotype in WT mice beginning at 5 weeks. Mice were subjected to ledge, clasping, gait, and kyphosis tests, and performance in each test was scored on a scale of 0–3. The average composite scores for each genotype at different ages were calculated. Bars represent SEMs. *p < 0.05 and **p < 0.01, compared with the WT group

Relative to WT littermates, NPC1^−/−^ mice were smaller at weaning and exhibited an initial weight loss at 7 weeks of age (Fig. 1A), decreased rotarod time (Fig. 1B), impaired coat hanger performance (Fig. 1C), and a shortened life span of 65.3 ± 3.6 days (Fig. 1D). These pathological phenotypes are consistent with previous reports [27]. To evaluate the relationship between neurological phenotype and onset time in NPC1^−/−^ mice, we used a composite phenotype scoring system based on sensitive and rapid quantification of disease severity [21]. The NPC1^−/−^ mice exhibited progressive cerebellar ataxia starting at 5 weeks of age (Figs. 1E, S1).

### Overview of the lncRNA-seq and mRNA-seq Data

RNA-seq analysis of lncRNA and mRNA expression levels in the cerebella of NPC1^−/−^ and WT mice generated 431,617,074 raw reads. Of these, 218,165,940 raw reads were from the WT mice and 213,451,134 were from the NPC1^−/−^ mice. Upon discarding low-quality sequences, adapter sequences, or sequences for which poly-N > 10%, 430,631,162 clean reads remained. Of these, 217,672,324 were from the WT mice and 212,958,838 were from the NPC1^−/−^ mice. Next, we conducted a comparative analysis based on paired-end clean reads in the reference genome using HISAT2 [28]. Coding-Non-Coding-Index (CNCI) (v2) [29] and Coding Potential Calculator (CPC) [30] were used to exclude (filter out) transcripts with predicted coding potential. A total of 30,779 lncRNAs (30,196 known lncRNAs and 583 novel lncRNAs) and 22,287 protein-coding transcripts (mRNAs) were then used for subsequent analyses. The identified lncRNAs included bidirectional, intergenic, intronic, antisense-overlapping, and sense-overlapping lncRNAs (Figure S2).

### DE lncRNAs and mRNAs in NPC1^−/−^ Mice Compared to WT Mice

**Fig. 2:**
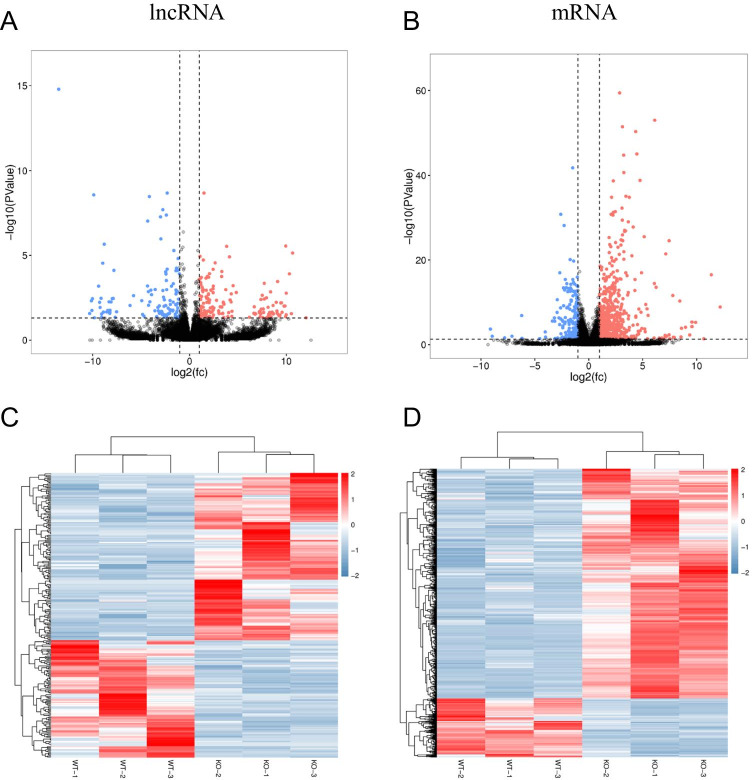
Expression patterns of lncRNAs and mRNAs in WT and NPC1^−/−^ mice. Volcano plots of DE lncRNAs (**A**) and mRNAs (**B**). Blue, red, and black points represent lncRNAs or mRNAs that were downregulated, upregulated, or not significantly different in NPC1^−/−^ (KO) mice relative to WT mice. Heatmap of DE lncRNAs (**C**) and mRNAs (**D**). Red and blue: increased and decreased expression, respectively

Overall, 272 lncRNAs and 856 mRNAs were found to be remarkably dysregulated in the NPC1^−/−^ mice (fold change ≥ 2.0, *p* < 0.05). Of these, 160 lncRNAs and 682 mRNAs were significantly upregulated, while 112 lncRNAs and 174 mRNAs were significantly downregulated in the NPC1^−/−^ mice versus the WT mice. Thirty lncRNAs and 4 mRNAs were exclusively expressed in the WT mice, while 55 lncRNAs and 10 mRNAs were exclusively expressed in the NPC1^−/−^ mice. When the NPC1^−/−^ mice were compared with the WT mice, the most significantly upregulated lncRNAs and mRNAs were Trem2 (14-fold change) and Clec7a (36-fold change), while the most significantly downregulated lncRNAs and mRNAs were Eps8l2 (8-fold change) and Svil (9-fold change). The top 10 most significantly DE lncRNAs and mRNAs are shown in Tables 1 and 2, respectively. Volcano plots and cluster analyses indicated that the lncRNA and mRNA expression patterns were variable and distinguishable between the groups of mice (Fig. 2A–D).

**Table 1:** Top 10 upregulated and 10 downregulated lncRNAs

Ensembl gene ID	Symbol	WT-1	WT-2	WT-3	KO-1	KO-2	KO-3	Fold change	*p* value	Regulation
ENSMUST00000148545	Trem2	3.7004	1.0000	1.0000	6.7142	4.8580	6.5392	14.3121	0.0000029	Up
ENSMUST00000142962	Gm15631	2.8074	3.1699	1.0000	5.2095	4.9069	6.4594	10.0690	0.0000889	Up
ENSMUST00000031975	Clec5a	1.0000	2.5850	1.0000	5.3219	4.0000	5.1699	9.9600	0.0001768	Up
ENSMUST00000195685	A330015K06Rik	3.5850	3.3219	3.4594	6.6724	4.7549	6.1898	6.2609	0.0000273	Up
ENSMUST00000136359	H19	4.3219	4.8580	5.9069	8.3264	7.3219	6.5236	5.4498	0.0000664	Up
ENSMUST00000166109	Eci2	2.3219	2.5850	2.0000	4.9542	4.1699	4.7549	5.4028	0.0015623	Up
ENSMUST00000131025	Uap1l1	3.8074	2.0000	2.5850	5.7004	4.5850	5.4594	5.3537	0.0009304	Up
ENSMUST00000128338	Btbd11	3.8074	1.0000	3.5850	5.2854	5.2095	5.1699	4.5405	0.023154	Up
ENSMUST00000235045	Gm2629	3.5850	4.0875	3.8074	5.9307	5.0444	6.3750	4.1694	0.0001185	Up
ENSMUST00000135230	Copg2	6.8455	5.2479	4.7549	7.1898	7.4998	7.7748	3.3397	0.0011418	Up
ENSMUST00000155729	Eps8l2	6.0000	6.3219	5.9542	3.5850	2.8074	2.3219	7.9545	5.34E-08	Down
MSTRG.20510.2	-	7.1898	7.9715	6.1293	2.0000	4.5236	4.8580	7.7788	1.06E-06	Down
ENSMUST00000126572	Gm13944	4.3219	5.8580	4.3219	1.0000	2.8074	2.3219	6.7802	0.0011752	Down
ENSMUST00000138576	Fgf7	5.9773	6.6147	5.8580	4.3219	3.5850	2.0000	5.5377	9.37E-05	Down
ENSMUST00000238391	Rian	5.3219	7.4594	5.3576	4.6439	1.0000	4.4594	5.5229	0.0090995	Down
ENSMUST00000152283	Gipc2	5.1293	4.8580	5.5236	2.8074	2.3219	3.0000	5.3684	0.0001263	Down
ENSMUST00000139218	Gm16201	6.2288	6.1699	6.2479	3.7004	3.7004	3.8074	5.2619	4.16E-08	Down
ENSMUST00000150330	Necap2	5.2095	4.9542	5.1699	1.5850	2.8074	3.3219	5.2566	0.0003048	Down
ENSMUST00000181447	D430036J16Rik	5.2479	5.6439	5.3219	2.0000	3.1699	3.4594	4.9706	6.52E-05	Down
MSTRG.13285.1	-	9.2715	9.8611	9.3729	6.0000	7.6582	7.1599	4.9444	2.11E-09	Down

**Table 2:** Top 10 upregulated and 10 downregulated mRNAs

Ensembl gene ID	Gene symbol	WT-1	WT-2	WT-3	KO-1	KO-2	KO-3	Fold change	*p* value	Regulation
ENSMUSG00000079293	Clec7a	3.0000	4.9069	3.4594	9.3859	7.9189	9.2621	35.5133	3.08E-26	Up
ENSMUSG00000024672	Ms4a7	1.5850	2.0000	2.0000	7.2854	6.0444	6.9307	29.8605	1.05E-17	Up
ENSMUSG00000018774	Cd68	4.3219	3.7004	3.8074	9.0848	7.7814	9.1472	27.0776	1.52E-39	Up
ENSMUSG00000038147	Cd84	5.3576	5.6724	5.5850	9.9410	8.7879	9.9773	22.1628	8.68E-46	Up
ENSMUSG00000069516	Lyz2	8.5584	8.8106	8.4757	13.3692	11.8234	13.1991	20.4264	4.62E-51	Up
ENSMUSG00000035273	Hpse	2.5850	5.0000	4.0875	8.8826	7.3309	8.7347	19.5488	3.84E-16	Up
ENSMUSG00000071713	Csf2rb	4.1699	3.4594	2.8074	6.6582	6.0224	6.8580	19.3250	1.58E-13	Up
ENSMUSG00000050335	Lgals3	4.5236	5.2479	4.8580	9.6073	8.1699	8.9773	17.4630	1.63E-28	Up
ENSMUSG00000004552	Ctse	3.9069	4.1699	2.5850	8.0279	7.0661	7.5157	15.9878	9.87E-22	Up
ENSMUSG00000040552	C3ar1	5.4263	5.8074	5.6439	9.7715	8.5584	9.5058	13.6313	1.45E-35	Up
ENSMUSG00000024236	Svil	6.1699	7.3663	8.1997	2.3219	4.8074	3.9069	9.0652	4.60E-07	Down
ENSMUSG00000041261	Car8	13.7355	14.6258	14.0700	9.9054	11.6041	10.6671	8.8879	3.06E-06	Down
ENSMUSG00000041476	Smpx	5.8074	6.3923	6.0224	2.0000	3.5850	2.3219	7.6154	2.30E-08	Down
ENSMUSG00000027208	Fgf7	7.4838	8.9218	8.4471	5.0000	5.6147	4.4594	7.5887	2.28E-11	Down
ENSMUSG00000002930	Ppp1r17	11.0573	11.5920	11.0471	7.4676	8.9687	8.2240	6.7945	4.90E-07	Down
ENSMUSG00000027296	Itpka	9.2574	9.6883	8.9278	5.9307	6.8826	6.5699	6.4412	1.08E-17	Down
ENSMUSG00000022686	B3gnt5	8.7715	9.9054	9.2119	6.3750	7.3837	6.6865	6.1338	2.13E-09	Down
ENSMUSG00000024413	Npc1	10.3509	10.7805	10.7764	8.1749	7.6795	8.1548	6.0209	1.55E-31	Down
ENSMUSG00000054901	Arhgef33	8.7177	9.4959	8.8486	4.4594	7.2668	5.5546	5.9614	0.002785	Down
ENSMUSG00000028222	Calb1	14.0292	14.7406	14.2434	11.0154	12.2761	11.8078	5.5053	1.64E-06	Down

### Validation of Gene Expression Profiles by qRT-PCR

**Fig. 3:**
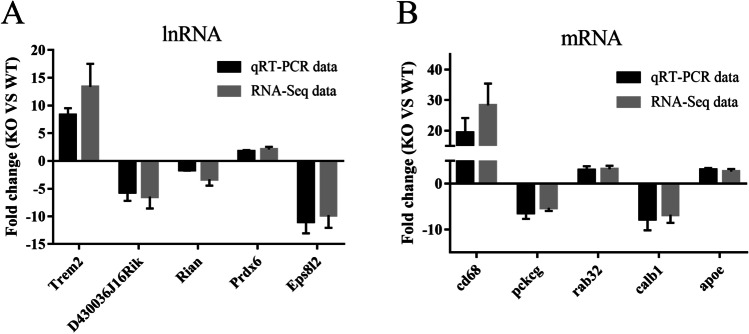
The differential expression of 5 randomly selected lncRNAs (**A**) and 5 randomly selected mRNAs (**B**) was validated by qRT-PCR

To verify the reliability and accuracy of the differential expression profiles identified by RNA-seq, 5 lncRNAs (Trem2, D430036J16Rik, Rian, Prdx6, and Eps8l2) and 5 mRNAs (cd68, pckcg, rab32, calb1, and apoe) were randomly selected for qRT-PCR validation. All selected lncRNA and mRNA transcripts were detected with significantly different expressed by qRT-PCR, consistent with the RNA-seq data (Fig. 3A–B).

### lncRNA-mRNA Network Analysis in NP-C

**Fig. 4:**
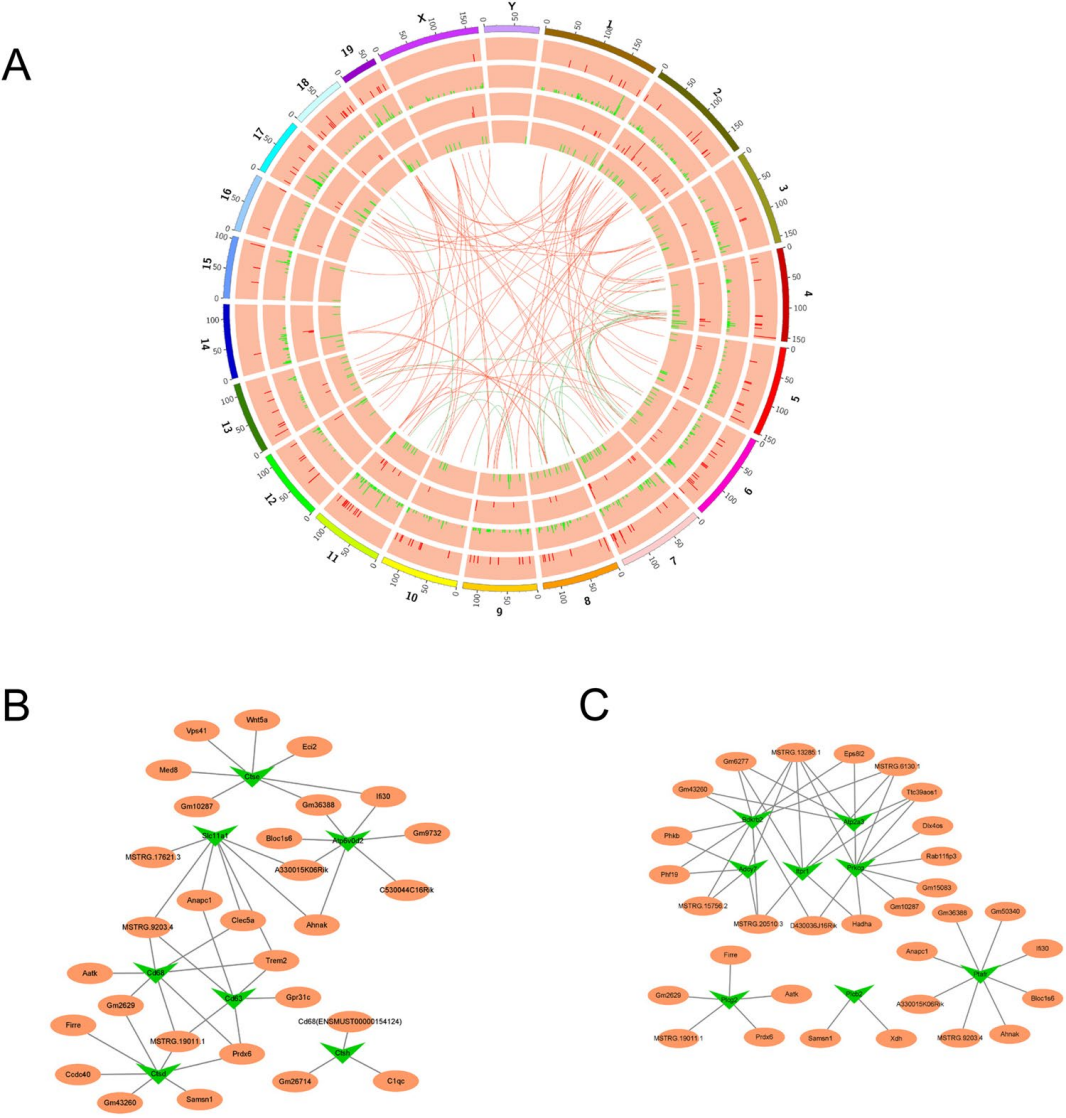
LncRNA-mRNA coexpression network analysis. **A** Circos diagram of DE lncRNAs and mRNAs. The outermost circle is a schematic of the murine chromosome distribution. The second and third circles (from the outermost circle) represent the chromosomal distribution of DE mRNAs. The fourth and fifth circles represent the chromosomal distribution of DE lncRNAs. Red and green lines indicate up- and down-regulation, respectively. Internal connections indicate the top 100 strongest correlations between lncRNAs and mRNAs. **B** Coexpression network of lysosome (Lys)–related genes and lncRNAs. **C** Coexpression network of calcium-related genes and lncRNAs. Orange and green nodes represent dysregulated lncRNAs and mRNAs, respectively

To elucidate the potential roles of key lncRNAs and interactions between DE lncRNAs and mRNAs, we built a lncRNA-mRNA coexpression network based on a previously described analytical strategy [31]. The coexpression network consisted of 970 network nodes and 3318 correlations between 738 DE mRNAs and 232 DE lncRNAs. The network contained 725 negative and 2594 positive interactions, suggesting that a single lncRNA can interact with multiple mRNAs and vice versa (Table S3). The DE lncRNAs were mainly distributed on chromosomes 2, 7, and 4 (Fig. 4A), while the DE mRNAs were mainly distributed on chromosomes 11, 6, and 1 (Fig. 4A). The internal connections on the Circos diagram indicate the top 100 strongest correlations between lncRNAs and mRNAs.

Mounting evidence indicates that impaired lysosomal function and calcium distribution in the cellular reticular network mediate NP-C pathogenesis [32, 33]. Thus, Lys- and calcium-related genes were incorporated into the coexpression network (Fig. 4B–C). Potential interactions between lncRNAs and mRNAs appear to mediate the development of NP-C.

### GO and KEGG Pathway Analyses

**Fig. 5:**
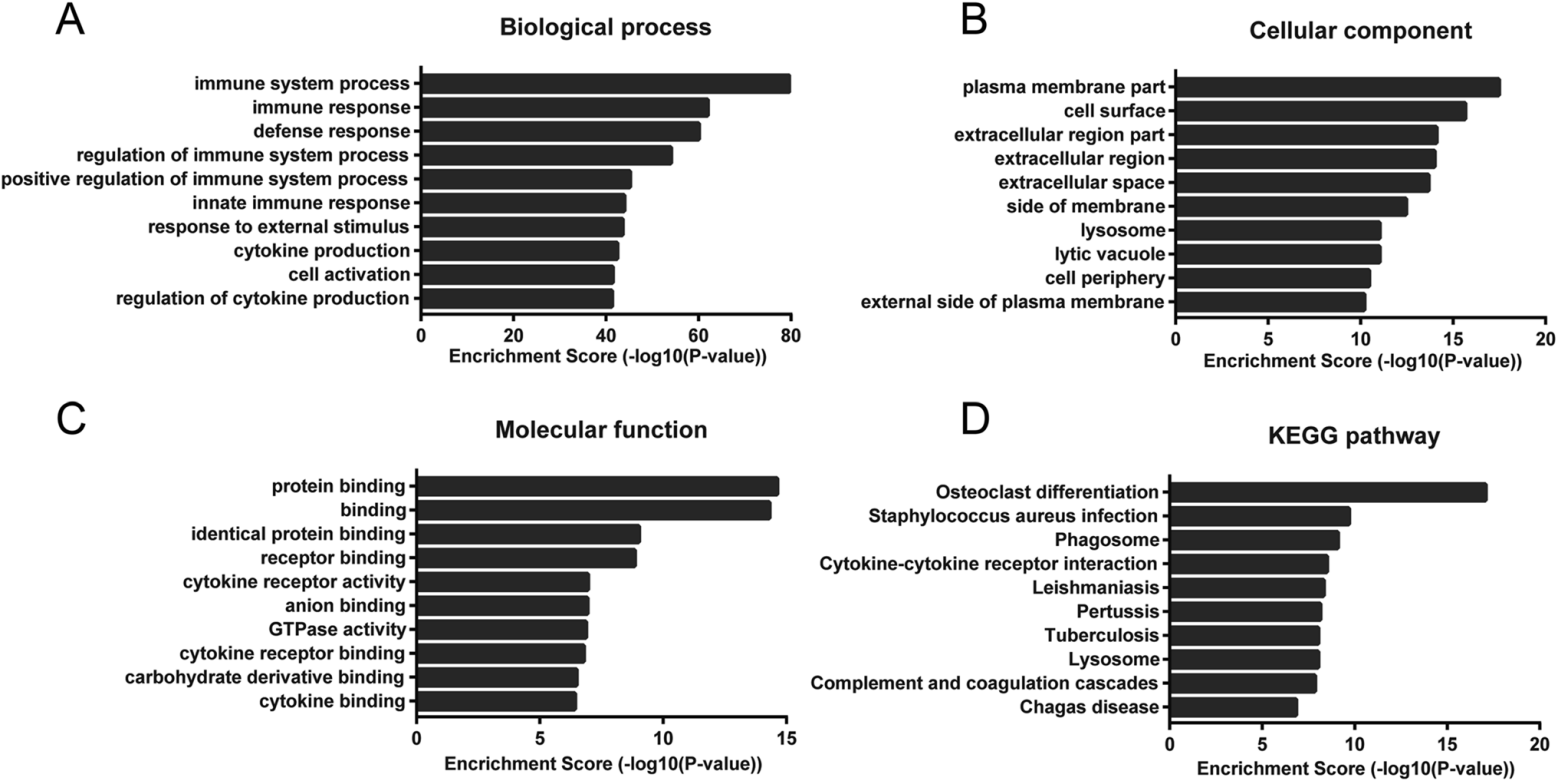
GO enrichment and KEGG pathway analyses of DE genes in NPC1^−/−^ mice versus WT mice. **A** BP, **B** CC, **C** MF, and **D** KEGG pathways enriched in the significantly DE genes; the top 10 most significant terms from the enrichment analysis are presented (*p* < 0.05)

Next, we carried out GO and KEGG pathway analyses of the mRNAs in the coexpression network. The terms enriched in a given lncRNA indicated its predicted biological functions. GO analysis revealed that the mRNAs coexpressed with lncRNAs were mainly linked to the immune system process (biological process, BP), plasma membrane (cellular component, CC), and protein binding (molecular function, MF) (Fig. 5A–C). KEGG pathway analysis identified osteoclast differentiation, *Staphylococcus* *aureus* infection, and phagosomes as the most enriched pathways (Fig. 5D).

### NPC1-Related Coexpression Networks in NP-C

**Fig. 6:**
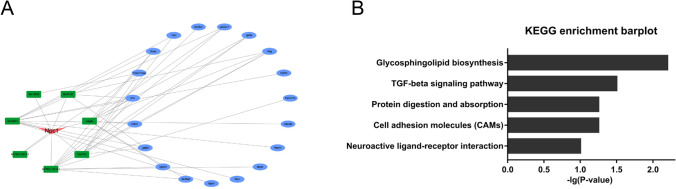
Construction of a NPC1-related coexpression network. **A** Green nodes represent lncRNAs that were significantly coexpressed with NPC1. Blue nodes represent coexpressed genes. **B** KEGG analysis indicated that NPC1-lncRNA-coexpressed mRNAs were mainly targeted to the glycosphingolipid biosynthesis pathway

NP-C evolves primarily due to mutations in the NPC1 gene; thus, further investigation of NPC1-lncRNA interactions may significantly enhance our understanding of NP-C. Here, we identified NPC1 coexpression with 7 lncRNAs. Next, mRNAs coexpressed with the 7 lncRNAs were used to construct a subnetwork (Fig. 6A). Examination of the KEGG pathway annotations for which *p* < 0.05 revealed that these genes may be involved in the glycosphingolipid biosynthesis, TGF-beta signaling, protein digestion and absorption, cell adhesion molecule, and neuroactive ligand-receptor interaction pathways (Fig. 6B).

### Dysregulated Expression of the lncRNA H19 in NP-C

**Fig. 7:**
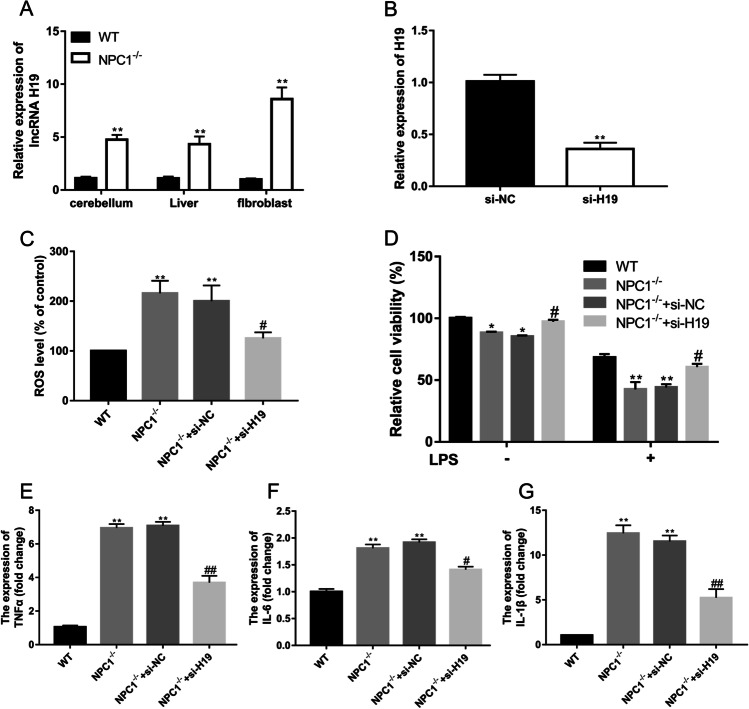
The effect of lncRNA H19 knockdown on oxidative stress. **A** Expression of H19 in cerebellar, liver and skin fibroblasts of mice. **B** H19 expression was evaluated after electroporation of H19-siRNA in skin fibroblasts. **C** ROS generation was detected by CM-H_2_DCFDA staining. **D** The viability of the fibroblasts that treated with or without 100 ng/mL LPS for 24 h was detected by CCK-8 assay. The expression levels of the inflammatory cytokines TNF-a (E), IL-6 (F), and IL-1β (G) after 24 h of stimulation with 100 ng/mL LPS were measured by qRT-PCR. Data are the mean ± SEM from three independent experiments. *p < 0.05 and **p < 0.01, compared with the WT group; #*p* < 0.05 and ##*p* < 0.01, compared with NPC1^−/−^  + si-NC group

Among the top dysregulated lncRNAs (Table 1), we focused on upregulation of the lncRNA H19 in NPC1^−/−^ mice. We first validated the dysregulated expression of the lncRNA H19 in cerebellar and liver tissues (Fig. 7A). Increased expression of the lncRNA H19 has been shown to be closely associated with inflammation [32–35]. Next, we explored whether the dysregulated lncRNA H19 was involved in the inflammatory response in vitro. Expression of the lncRNA H19 was significantly increased in skin fibroblasts derived from the NPC1^−/−^ mice compared to the WT mice (Fig. 7A). We then used siRNA to successfully knock down H19 in NPC1^−/−^ fibroblasts (Fig. 7B). A significantly decreased ROS level was detected in the NPC1^−/−^ fibroblasts after knockdown of the lncRNA H19 (Fig. 7C). Moreover, knockdown of the lncRNA H19 reversed the change in viability of the NPC1^−/−^ fibroblasts treated with or without LPS, whose viability was similar to that of the WT fibroblasts (Fig. 7D). TNF-α, IL-6, and IL-1*β*, as proinflammatory cytokines, promote the inflammatory response, and their expression can be induced by inflammatory activators such as LPS*.* H19 knockdown attenuated LPS-induced expression of these proinflammatory cytokines in the NPC1^−/−^ fibroblasts (Fig. 7E–G). Taken together, these data suggested that lowering H19 expression could be a strategy to ameliorate oxidative and inflammatory damage in NP-C.

## Discussion

Dysregulated lncRNAs have been suggested to play important roles in the pathological processes of numerous neurodegenerative neurological disorders, particularly neuroinflammation, the modulation of Aβ enrichment/production, synaptic transmission, neurotrophin depletion, and mitochondrial dysfunction [36]. The best-known example of a lncRNA is β-secretase-1 antisense RNA (BACE1-AS) [12], which drives formation of Alzheimer’s disease–implicated forms of Aβ peptides [37]. NP-C is a typical neurodegenerative disorder sometimes referred to as childhood Alzheimer’s disease. However, the pathological implications of lncRNAs in NP-C remain undetermined. Cerebellar ataxia, a major hallmark of NP-C disease, is mainly caused by the progressive degeneration of Purkinje cells [6, 8, 38]. Here, we applied RNA-seq analysis to profile the expression patterns of lncRNAs and mRNAs in cerebellar tissue from NPC1^−/−^ mice and uncovered 160 lncRNAs and 682 mRNAs as significantly upregulated and 112 lncRNAs and 174 mRNAs as significantly downregulated compared to their expression in WT littermates (FC ≥ 2.0, *p* < 0.05). The identification of these dysregulated lncRNAs can provide insight into the investigation of novel mechanisms underlying the pathological processes of NP-C.

Given that anomalous lysosomal function and Ca^2+^ signaling play central roles in NP-C pathology [32, 33], we explored the association between DE lncRNAs and Lys- and calcium-associated genes. We also constructed a NPC1-lncRNA coexpression network and added the mRNAs coexpressed with 7 lncRNAs for pathway annotation analysis. KEGG analysis suggested that genes coexpressed with 7 lncRNAs are mainly enriched in the glycosphingolipid biosynthesis pathway. The accumulation of a massive amount of glycosphingolipid in the neurosystem was demonstrated to be a major feature of NP-C [39]. Currently, miglustat, a glycosphingolipid biosynthesis inhibitor, is the only medication approved for NP-C patients in some areas and countries, including the EU and China [40, 41]. Miglustat administration was found to delay neurological dysfunction onset and extend average life span in NP-C animal models. Moreover, miglustat treatment improved clinical symptoms and quality of life in NP-C patients. However, miglustat was originally developed and applied for Gaucher disease, and its application was extended for NP-C disease. The mechanisms underlying miglustat treatment for NP-C are not clear. Therefore, its clinical application in NP-C patients has not been approved by the FDA in the USA. The dysregulated lncRNAs revealed to be related to impaired glycosphingolipid metabolism due to *NPC1* mutation have great potential for the development of novel medications targeting the glycosphingolipid biosynthesis pathway.

GO and KEGG pathway analyses were performed to identify coding genes related to the significantly dysregulated lncRNAs. GO analysis showed that the enrichment of biological processes such as the immune system process, immune response, defense response, and innate immune response, which have been implicated in neurodegenerative diseases and cognitive dysfunction, in these lncRNAs. Neuroinflammation, a common pathological hallmark of most neurodegenerative diseases, influences neuronal development and function [42]. NP-C patients and NPC1^−/−^ mice exhibit abnormal mitochondrial function and increased oxidative stress [43, 44]. In our study, we also observed that ROS level significantly increased in the NPC1^−/−^ fibroblasts. Therefore, we suggest that pathological inflammation not only actively contributes to NP-C pathogenesis but also is a potential therapeutic target in NP-C.

The lncRNA H19, which is involved in immune and inflammatory responses, promotes microglia and astrocyte activation under epileptic and normal conditions [45]. In addition, the lncRNA H19 enhances neuroinflammation by driving HDAC1-dependent microglial M1 polarization during ischemic stroke [46]. Here, we found that levels of the lncRNA H19 were significantly upregulated in the cerebellar, liver and skin fibroblasts of NPC1^−/−^ mice. Silencing the lncRNA H19 in skin fibroblasts ameliorated the changes in ROS levels and cell viability and inflammatory response induced by LPS, suggesting that inhibition of the lncRNA H19 may improve the pathological features of NP-C disease via inflammatory modulation.

Some of the other dysregulated lncRNAs that we identified have been reported to be involved in pathological processes in other neurological diseases. The lncRNA Neat1 was found to be significantly upregulated in the caudate nucleus in Huntington’s disease [47] and plays an important role in innate immunity [48]. In an ischemic stroke rat model, the lncRNA MIAT could induce the autophagy and apoptosis of neural cells [49]. The lncRNA Rian was decreased in a model of cerebral ischemia–reperfusion injury, and overexpression of the lncRNA Rian significantly reduced infarct size and improved neurological function score [50]. These findings suggest that the aberrant expression of lncRNAs may have similar effects in the above neurological diseases and NP-C.

There are several interesting issues that remain to be addressed. First, we report the profiles of only lncRNAs in the cerebellum, and the expression patterns of lncRNAs in the blood and cerebral spinal fluid in NP-C remain to be determined. Furthermore, it is unclear whether the significantly dysregulated lncRNAs can serve as diagnostic biomarkers for NP-C. Second, we predicted the functions of DE lncRNAs through bioinformatics analysis of mRNAs coexpressed with the lncRNAs; however, it is unclear whether these lncRNAs would modulate expression of the corresponding coding genes in vitro or in vivo. Third, determining the spatiotemporal expression patterns of the DE lncRNAs and mRNAs requires further measurements to more precisely reflect the pathophysiology of NP-C.

## Conclusion

This study has, for the first time, determined the expression patterns of lncRNAs in a NP-C mouse model. Our results show that aberrantly expressed lncRNAs are involved in various pathological processes, especially immune system–related processes, and we demonstrated that the lncRNA H19 was associated with the inflammatory response in vitro. These findings provide new insights into NP-C pathogenesis and unveil novel therapeutic targets.

## Supplementary Information

Below is the link to the electronic supplementary material.

Supplementary file1 (DOCX 17 KB)

Supplementary file2 (XLSX 180 KB)

Supplementary file3 (PDF 305 KB)

## Data Availability

The RNA-seq raw data are available on Sequence Read Archive (SRA) under the accession number PRJNA682842. All animal experimental methods performed were conducted in accordance with the ethical standards and procedures of the ethics committee approved by the Army Medical University (Chongqing, China) under permit NO. amuwec20181550.
