# Supplementary material for: Cerebellar Long Noncoding RNA Expression Profile in a Niemann-Pick C Disease Mouse Model
Source: Mol Neurobiol. 2021 Aug 19;58(11):5826–36. doi: 10.1007/s12035-021-02526-3 (PMC8599378; doi:10.1007/s12035-021-02526-3)
Supplement: Supplementary file 1 — Supplementary file1 (DOCX 17 KB) [file 12035_2021_2526_MOESM1_ESM.docx]

**Table S1.** The RNA-seq profile for GAPDH

| **Ensembl gene ID** | **Symbol** | **WT-1** | **WT-2** | **WT-3** | **KO-1** | **KO-2** | **KO-3** |
| --- | --- | --- | --- | --- | --- | --- | --- |
| ENSMUSG00000057666 | Gapdh | 1290.56 | 1060.47 | 978.25 | 1101.4 | 1159.28 | 1099.88 |

**Table S2.** Primers designed for qRT-PCR validation of candidate lncRNAs and mRNAs.

|  | **Forward primer** | **Reverse primer** |
| --- | --- | --- |
| **Trem2** | CTGCTGGCCTTCCTGAAGAA | TCGGAGACTCTGACACTGGT |
| **D430036J16Rik** | TTGGGCTGTGGTGAGTTCTC | GAGGACCAAGATGGGGCAAA |
| **Rian** | AGAACCAGCAGCCCAGAATC | CCAGATTTCAGGGTGGCAGT |
| **Prdx6** | GCATCCGCTTCCACGATTTC | TGCACACTGGGGTAAAGTCC |
| **Eps8l2** | GAAGGCCTTCCTGGAGAAACA | GTACAGCGGATGCAGGAAAAC |
| **cd68** | GAAATGTCACAGTTCACACCAG | GGATCTTGGACTAGTAGCAGTG |
| **Prkcg** | GCTCCGACGAACTCTATGCCATC | CCAATGCCAGGACACGCTTCTC |
| **rab32** | GGCAACATGACTCGAGTATACT | CCGTTGGGAAGATGAACTTTAC |
| **calb1** | TCGAAACCGAGGAACTTAAGAA | GGTCTGTGTACTCTGCTAGTTT |
| **apoe** | GAGGAACAGACCCAGCAAATA | CGATGCATGTCTTCCACTATTG |
| **TNF-α** | CTGAACTTCGGGGTGATCGG | GGCTTGTCACTCGAATTTTGAGA |
| **IL-6** | CTGCAAGAGACTTCCATCCAG | AGTGGTATAGACAGGTCTGTTGG |
| **IL-1β** | GAAATGCCACCTTTTGACAGTG | TGGATGCTCTCATCAGGACAG |
| **GAPDH** | TGTGTCCGTCGTGGATCTGA | CCTGCTTCACCACCTTCTTGA |
