# Supplementary material for: Cerebellar Long Noncoding RNA Expression Profile in a Niemann-Pick C Disease Mouse Model
Source: Mol Neurobiol. 2021 Aug 19;58(11):5826–36. doi: 10.1007/s12035-021-02526-3 (PMC8599378; doi:10.1007/s12035-021-02526-3)
Supplement: Supplementary file 3 — Supplementary file3 (PDF 305 KB) [file 12035_2021_2526_MOESM3_ESM.pdf]

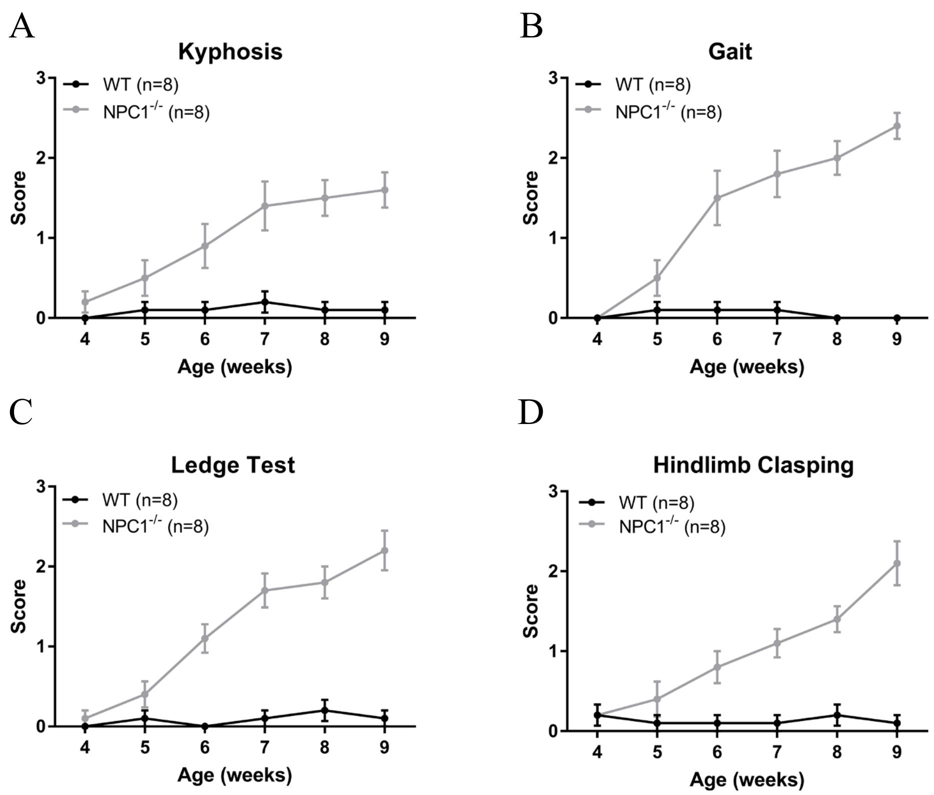

Figure S1. Neurobehavioral assessment of WT and NPC1$^{-/-}$ mice. The assessment involved kyphosis tests (A), gait (B), ledge test (C) and hindlimb clasping (D). Each measure was assessed on a scale of 0-3, with 0 indicating no phenotype, 1 indicating weak phenotype, 2 indicating strong phenotype and 3 indicating the most severe phenotype, for a total score of 0-12.

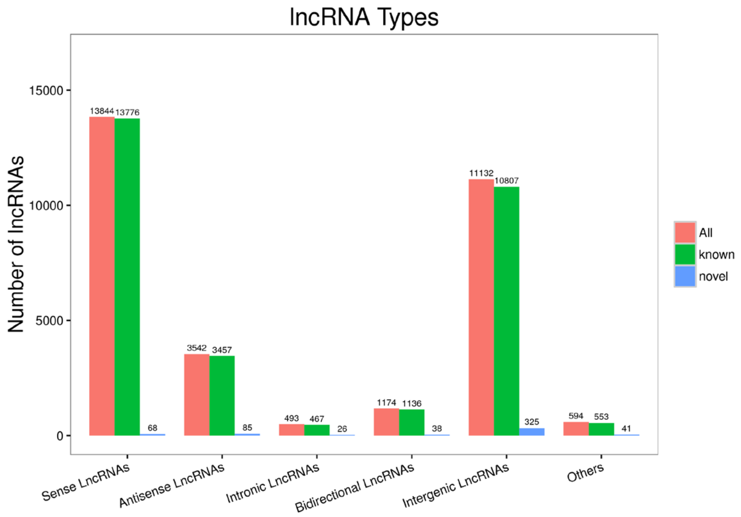

Figure S2. Classification of lncRNAs according to localization.
